# Shift in the Intrinsic Excitability of Medial Prefrontal Cortex Neurons following Training in Impulse Control and Cued-Responding Tasks

**DOI:** 10.1371/journal.pone.0023885

**Published:** 2011-08-22

**Authors:** Scott J. Hayton, Mary C. Olmstead, Éric C. Dumont

**Affiliations:** 1 Department of Psychology, Queen's University, Kingston, Ontario, Canada; 2 Centre for Neuroscience Studies, Queen's University, Kingston, Ontario, Canada; 3 Department of Anesthesiology and Perioperative Medicine, Queen's University, Kingston, Ontario, Canada; University of New South Wales, Australia

## Abstract

Impulse control is an executive process that allows animals to inhibit their actions until an appropriate time. Previously, we reported that learning a simple response inhibition task increases AMPA currents at excitatory synapses in the prelimbic region of the medial prefrontal cortex (mPFC). Here, we examined whether modifications to intrinsic excitability occurred alongside the synaptic changes. To that end, we trained rats to obtain a food reward in a response inhibition task by withhold responding on a lever until they were signaled to respond. We then measured excitability, using whole-cell patch clamp recordings in brain slices, by quantifying action potentials generated by the injection of depolarizing current steps. Training in this task depressed the excitability of layer V pyramidal neurons of the prelimbic, but not infralimbic, region of the mPFC relative to behavioral controls. This decrease in maximum spiking frequency was significantly correlated with performance on the final session of the task. This change in intrinsic excitability may represent a homeostatic mechanism counterbalancing increased excitatory synaptic inputs onto those neurons in trained rats. Interestingly, subjects trained with a cue that predicted imminent reward availability had increased excitability in infralimbic, but not the prelimbic, pyramidal neurons. This dissociation suggests that both prelimbic and infralimbic neurons are involved in directing action, but specialized for different types of information, inhibitory or anticipatory, respectively.

## Introduction

Effective interaction with the world around us depends on the ability to modify behavior in response to environmental cues. These cues provide information on appropriate actions, not only *which* responses are correct but also *when* they should be initiated. The selection and timing of responses are independent processes, in that a specific action may be neither right nor wrong, but require restraint until the appropriate moment, like waiting for a green light before crossing the street. Withholding a response during these periods is controlled by an executive process termed impulse control [1]. Impulse control provides a top-down signal to inhibit responses until a ‘Go’ signal is presented [2]. Unit recordings [3], lesions [4], and temporary inactivation [5], [6] all point to the medial prefrontal cortex (mPFC) as a critical neural substrate for impulse control. It is not surprising, therefore, that mPFC dysfunction and deficits in impulse control co-occur in many psychiatric disorders, including attention-deficit hyperactivity disorder [7], [8], drug addiction [1], [9], compulsive gambling [10], and binge eating [11].

Given its importance in impulse control, we investigated the mechanisms that underlie encoding response inhibition in the rat mPFC [12]. This work revealed that learning a simple response inhibition (RI) task increased the ratio of AMPA to NMDA currents in layer V pyramidal neurons of the prelimbic, but not infralimbic region of the mPFC. Notably, this enhancement to excitatory transmission was selective to neurons projecting to the ventral striatum. These changes, which resemble long-term potentiation [13], closely tracked performance in the RI task and suggested a mechanism for impulse control [12].

Alterations in synaptic transmission do not occur in isolation; compensatory mechanisms, either enhanced GABA transmission or decreased excitability, may be necessary to maintain neurons within their physiological firing rate range [14], [15]. In fact, the excitability of mPFC pyramidal neurons is modulated after training in fear conditioning and extinction [16], as well as cocaine withdrawal [17]–[19]. Thus, we hypothesized that the excitability of prelimbic neurons would be reduced to compensate for the increased glutamatergic transmission we reported previously [12].

To examine whether changes in intrinsic excitability in the mPFC correlate with impulse control, we trained rats in a simple RI task [12], [20]. This task requires subjects to wait for a short delay before pressing a lever to obtain a food reward. After learning to withhold responding, subjects were euthanized and brain slice whole-cell patch clamp recordings were made of layer V pyramidal neurons in the prelimbic and infralimbic regions of the mPFC. We then compared the excitability of these cells to untrained subjects and to subjects trained in one of four control versions of the RI task. Our study shows that training in this task depressed the excitability of layer V pyramidal neurons of the prelimbic, but not infralimbic, region of the mPFC relative to behavioral controls. These findings demonstrate that excitability and synaptic strength interact in the mPFC to direct impulse control.

## Results

### Learning Response Inhibition and Operant Control Tasks

**Figure 1:**
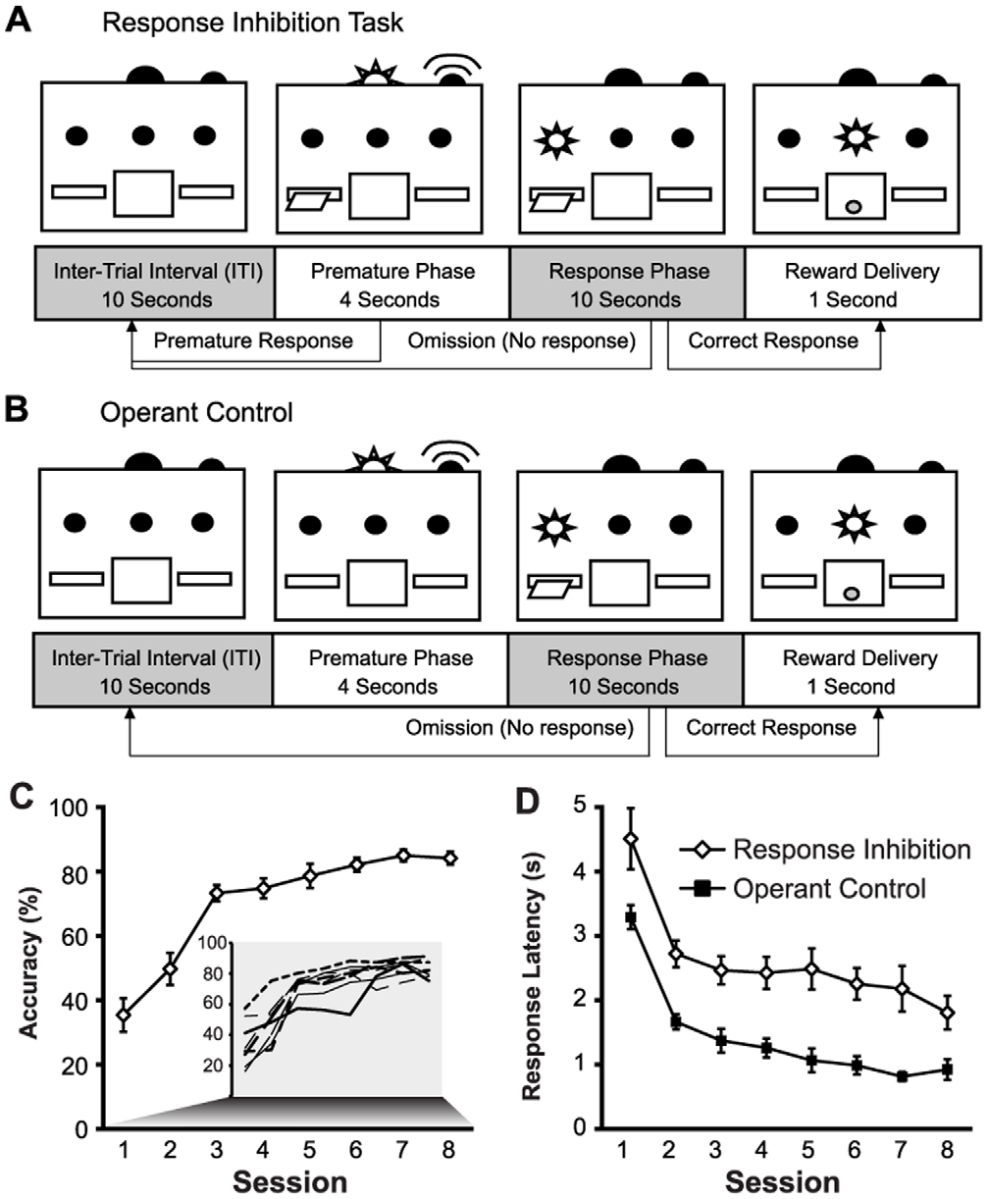
Behavioural training. **A.** The response inhibition (RI) task requires subjects to withhold responding until the correct phase. Responses during the correct phase result in a sucrose pellet reward and reinstate the intertrial interval (ITI). Responses during the premature phase restore the ITI with no reward. Failure to respond during the correct phase results in an omission and reinstates the ITI. **B.** The Operant Control condition is identical to the RI task, but the lever is withdrawn during the premature phase, preventing any premature responses. **C.** Accuracy rapidly improves over 8 sessions of training on the RI task (n = 8). **D.** Latency to respond decreases over training sessions on the RI (n = 8) and Operant control (n = 5) tasks. Response latencies were reduced in Operant Control subjects across all sessions.

Subjects rapidly learned the RI task (Figure 1C), improving accuracy across the eight training sessions (F_(1,7)_ = 38.81, p<0.001), although performance varied across individuals (Figure 1C inset). Subjects trained in the Operant Control task had shorter latencies to respond during the response phase than the RI group (Group: F_(1,13)_ = 38.86, p<0.001) (Figure 1D), and the time to respond decreased with training in both groups (Session: F_(7,91)_ = 25.10, p<0.001; Group x Session: F_(7,91)_ = 0.29, p = 0.96).

### Training Induced Changes in Prelimbic Neurons

**Figure 2:**
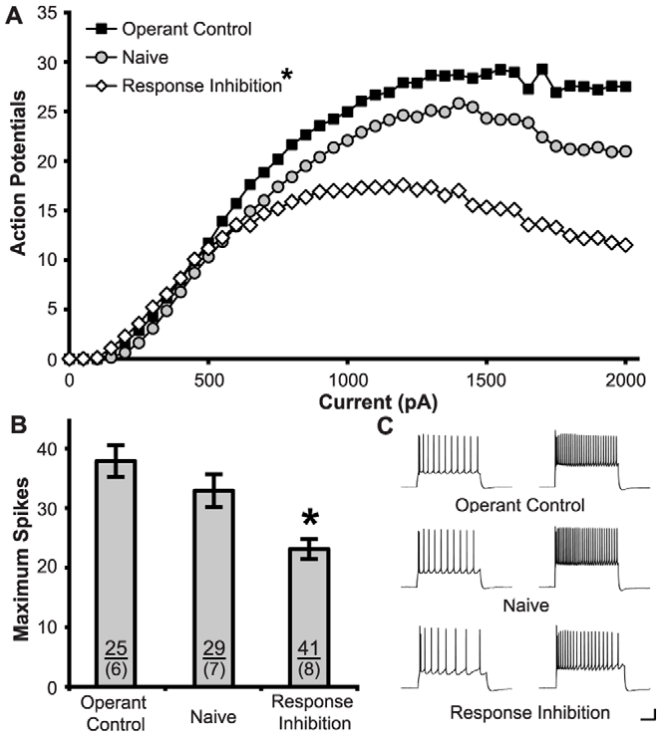
Training on the RI task reduced the intrinsic excitability of prelimbic neurons. **A.** Action potentials were produced by increasing current steps (800 ms, 50 pA steps). Learning the RI task produced less spiking than Operant Control and Naïve conditions. **B.** Learning the RI task decreased the maximum number of action potentials evoked. Sample sizes for subjects (above) and cells (below) are indicated within the bars. **C.** Representative traces show action potentials generated by 500 pA (left) and 1500 pA (right) current steps for RI, Operant Control, and Naïve subjects. (Calibration Bars: 20 mV/0.1 s; *: p<0.05 vs. all groups).

In prelimbic neurons (Figure 2), training in the RI task significantly modified the number of APs generated and ANOVA revealed effects of Current (F_(39,3588)_ = 121.13, p<0.001), Group (F_(2,92)_ = 11.35, p<0.001), and an interaction between Group and Current (F_(78,3588)_ = 7.091, p<0.001). Post-hoc tests confirmed a significant decrease in firing after training in the RI task, compared to the Operant (p<0.001) and Naïve (p<0.01) controls. Simple effects conducted at each current step revealed that training produced a significant decrease in spiking at all current steps greater than 600 pA (p<0.05). Learning the RI task also enhanced the maximum number of spikes evoked (F_(2,92)_ = 11.545, p<0.001) and post-hoc tests confirmed lower maximum spikes in RI subjects than Operant (p<0.001) and Naïve (p = 0.002) Controls.

Learning the RI task also had a significant effect on passive membrane properties (Table 1), significantly increasing the membrane resistance (F_(2,92)_ = 3.99, p = 0.02), although post-hoc tests showed this effect was significant when compared to Naïve subjects (p = 0.01) but not Operant Controls (p = 0.06). Simple effects conducted at each current step revealed that training in the RI task produced a significant increase in excitability (p<0.05) in response to small (150–200 pA) steps. Similarly, the minimum current required to generate an action potential (Rheobase) was also decreased after training in the RI task (F_(2,92)_ = 4.02, p<0.02). Finally, we observed no change in the firing threshold at rheobase (F_(2,92)_ = 1.34; p = 0.27).

**Table 1:** Characteristics of prelimbic neurons.

	Response Inhibition	Operant Control	Naïve Control
Resting Potential (Vm)	−67.79±0.71	−68.34±0.83	−68.32±0.67
Input Resistance (MΩ)	115.80±6.14 (#)	96.75±7.86	90.37±7.30
Rheobase (pA)	214.63±13.44 (*)	270.00±20.41	265.52±15.91
Maximum # Spikes	23.07±1.67 (*)	37.88±2.66	32.90±2.76
fAHP (mV)	−18.73±0.65	−17.69±0.63	−17.42±0.60
mAHP (mV)	−3.04±0.35	−3.45±0.27	−2.84±0.36
SAHP (mV)	−1.42±0.15	−1.13±0.13	−0.99±0.17

*: p<0.05 vs all groups;

# : p<0.05 vs naive.

**Figure 3:**
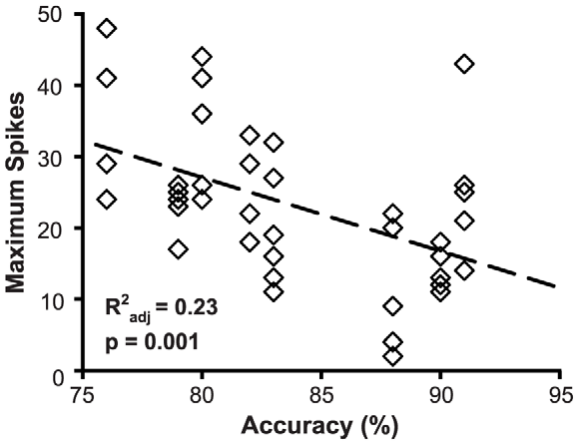
Performance of RI task predicts reduction in excitability. Plot depicts the negative relationship between accurate performance during the final training session of the RI task and maximum spikes evoked for individual prelimbic neurons. Hatched line shows linear regression.

To better understand the effect of training on excitability, a series of regressions were conducted to correlate accuracy on the subjects' final session of the RI task to electrophysiology measurements. This revealed that accuracy strongly predicted maximum spiking (R^2^_adj_ = 0.23, F_(1,40)_ = 12.76, p = 0.001) (Figure 3), but not input resistance (R^2^_adj_ = 0.00; F_(1,40)_ = 1.07; p = 0.31) or rheobase (R^2^_adj_ = 0.03; F_(1,40)_ = 2.09; p = 0.16).

Changes in excitability are frequently caused by changes in potassium channels, and can be measured by AHP. However, we observed no significant effect of training on fAHP (F_(2,74)_ = 1.09; p = 0.34), mAHP (F_(2,78)_ = 0.62; p = 0.54), or sAHP (F_(2,78)_ = 1.23; p = 0.30).

### Training Induced Changes in Infralimbic Neurons

**Figure 4:**
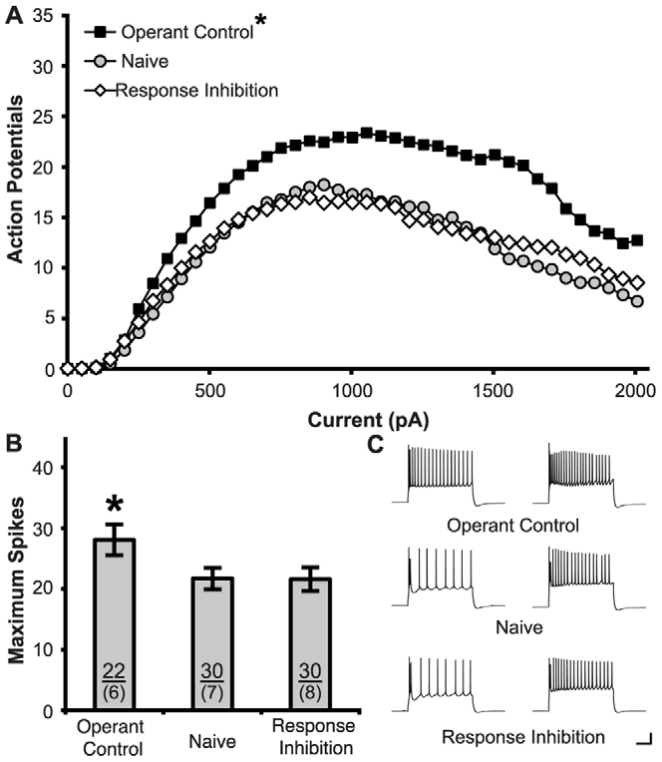
Training on the Operant Control condition increased the intrinsic excitability of infralimbic neurons. **A.** Action potentials were produced by increasing current steps (800 ms, 50 pA steps). Subjects in the Operant Control condition exhibited greater spiking than those in the RI and Naïve conditions. **B.** Learning the Operant Control task increased the maximum number of action potentials evoked. Sample sizes for subjects (above) and cells (below) are indicated within the bars. **C.** Representative traces show action potentials generated by 500 pA (left) and 1000 pA (right) current steps for RI, Operant Control, and Naïve subjects. (Calibration Bars: 20 mV/0.1 s; *: p<0.05 vs. all groups).

Training in the RI task failed to modulate excitability in the infralimbic region, but unexpectedly training in the Operant Control condition increased excitability (Figure 4A–B). ANOVA revealed a significant effect of Current (F_(39,3120)_ = 75.42, p<0.001) and Group (F_(2,79)_ = 5.84, p = 0.004), but no Current x Group interaction (F_(78,3120)_ = 1.70, p = 0.14). Post-hoc tests confirmed significantly more APs in neurons of the Operant Control group than in the Naïve (p = 0.002) and RI (p = 0.005) groups. Similarly, learning the Operant Control task also increased the maximum spikes observed (Figure 4C; F_(2,78)_ = 3.71, p<0.03). A regression showed that response latencies during the final training session for Operant Control subjects had no predictive relationship with maximum spiking (R2_adj_ = 0.05, F_(1,20)_ = 0.01, p = 0.93).

Passive membrane properties in the infralimbic region were unaffected by training in any of the tasks (Table 2). In contrast to prelimbic neurons, no effects of training were observed on input Resistance (F_(2,78)_ = 0.87) or rheobase (F_(2,78)_ = 0.38, p = 0.69). We also observed no effect of training on threshold to fire at rheobase (F_(2,78)_ = 2.36; p = 0.10).

**Table 2:** Characteristics of infralimbic neurons.

	Response Inhibition	Operant Control	Naïve Control
Resting Potential (Vm)	−65.00±0.68	−66.07±0.85	−65.94±0.75
Input Resistance (MΩ)	114.41±6.54	108.07±7.82	101.16±6.54
Rheobase (pA)	201.67±10.07	213.59±12.80	210.00±8.78
Maximum # Spikes	21.57±1.95	28.05±21.57 (*)	22.37±1.83
fAHP (mV)	−21.33±1.10	−19.31±1.01	−19.80±0.83
mAHP (mV)	−3.70±0.41	−4.54±0.35	−3.87±0.32
sAHP (mV)	−1.02±0.22	−1.07±0.21	−1.06±0.14

*: p<0.05 vs all groups.

No training-induced changes to AHP were observed in the infralimbic neurons. Specifically we saw no effect of training on fAHP (F_(2,65)_ = 1.22; p = 0.30), mAHP (F_(2,66)_ = 2.87; p = 0.06), or sAHP (F_(2,66)_ = 0.12; p = 0.89).

### Cue Induced Changes to mPFC Excitability

**Figure 5:**
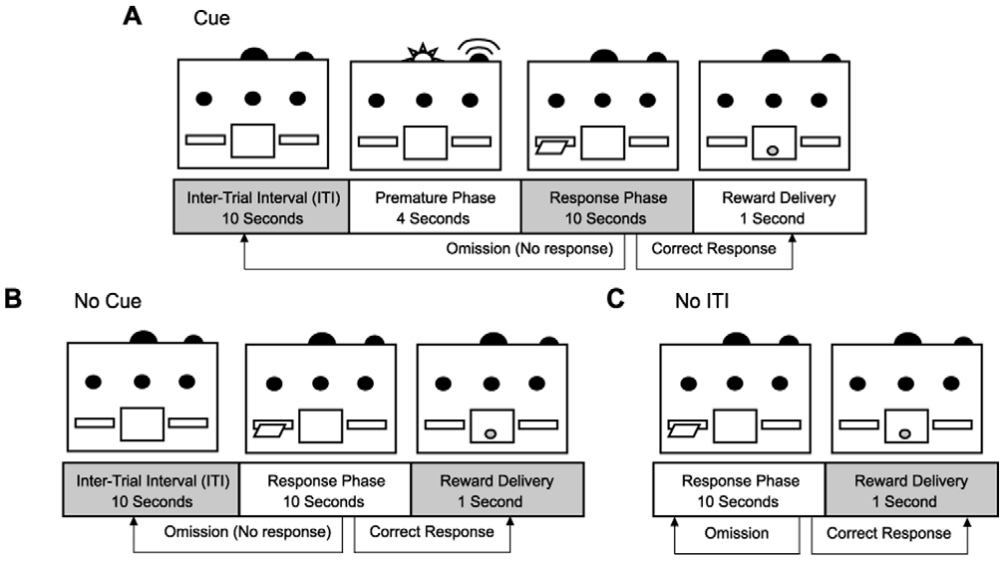
Behavioural training for three control conditions. **A.** The Cue condition is similar to the Operant Control condition, but the only stimuli are the sound/houselight during the premature phase. **B.** The ITI only condition had no premature phase, but the ITI was extended to 14 second to match the duration of the cue condition. **C.** The No ITI condition had no intertrial interval and, therefore, the lever was continuously available, except briefly (1 s) during reward delivery.

Training in the Operant Control task increased the excitability of infralimbic neurons. This condition exposed subjects to identical cues as the RI task, but the lever was withdrawn during the premature phase, preventing an incorrect response. Subjects in the Operant Control task, therefore, were presented with stimuli that predicted the imminent insertion of the lever. We hypothesized that repeated training with a cue prior to lever insertion was responsible for the changes we observed in this group. To address this possibility, we designed three additional tasks: the first (Cue) employed the cue but no other signals (Figure 5A); the second (No Cue) had no cue, but a matched ITI (Figure 5B); the third (No ITI) had no cue or ITI (Figure 5C). As previously, subjects were trained for 8 consecutive sessions, receiving 100 rewards per session.

**Figure 6:**
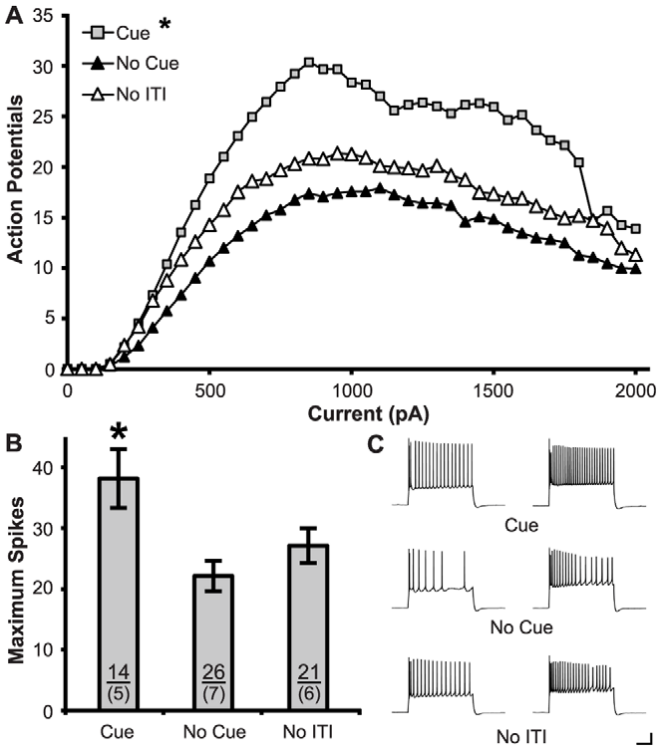
Training in the Cue condition increased the intrinsic excitability of infralimbic neurons. **A.** Action potentials were produced by increasing current steps (800 ms, 50 pA steps). Greater spiking occurred in the Cue condition than in the ITI only and No ITI conditions. **B.** Learning the cue increased the maximum number of action potentials evoked. Sample sizes for subjects (above) and cells (below) are indicated within the bars. **C.** Representative traces show action potentials generated by 500 pA (left) and 1000 pA (right) current steps for differing behavioral conditions. (Calibration Bars: 20 mV/0.1 s; *: p<0.05 vs. all groups).

Training with the cue increased the intrinsic excitability of infralimbic neurons (Figure 6). ANOVA revealed a significant effect of Current (F_(39,2262)_ = 46.03, p<0.001) and Group (F_(2,58)_ = 5.41, p<0.01) but no Group x Current interaction (F_(78,2262)_ = 1.25, p = 0.30). Post-hoc tests confirmed that subjects in the Cue condition had significantly more APs than the other two groups (p<0.05). Similarly, the maximum number of spikes also showed a significant effect of Group (F_(2,58)_ = 5.79, p = 0.005), with post-hoc tests confirming higher maximum spikes in the Cue group (p<0.05). Passive membrane properties were also unaffected by these conditions, with no effects observed on input resistance (F_(2,58)_ = 1.54, p = 0.22) or rheobase (F_(2,58)_ = 1.85, p = 0.17).

**Figure 7:**
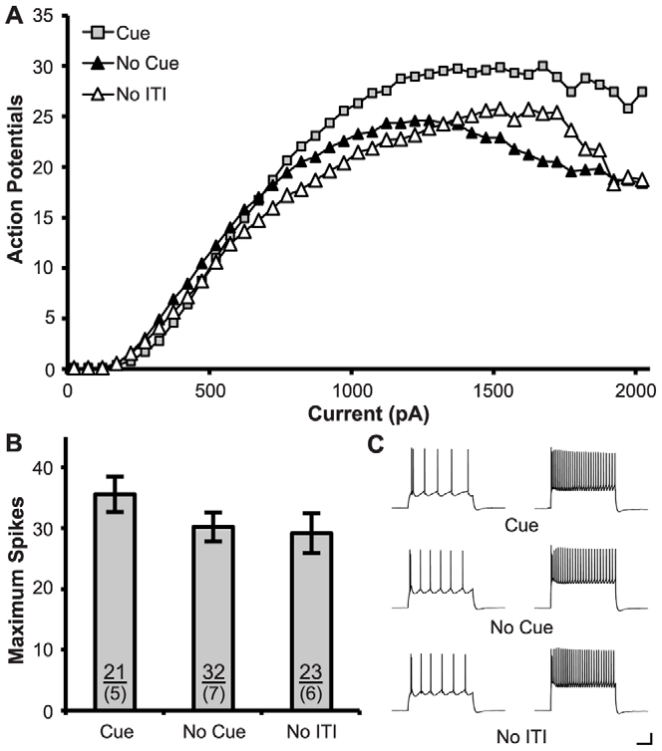
Training in the Cue condition had no effect on the intrinsic excitability of prelimbic neurons. **A.** Action potentials were produced by increasing current steps (800 ms, 50 pA steps). **B.** Learning the cue had no effect the maximum number of action potentials evoked. Sample sizes for subjects (above) and cells (below) are indicated within the bars. **C.** Representative traces show action potentials generated by 500 pA (left) and 1500 pA (right) current steps for differing behavioral conditions. (Calibration Bars: 20 mV/0.1 s; *: p<0.05 vs. all groups).

In prelimbic neurons, training with a cue failed to modulate intrinsic excitability (Figure 7). ANOVA revealed a significant effect of Current (F_(40,2920)_ = 94.12, p<0.001), but not Group (F_(2,73)_ = 1.51, p = 0.23) and no interaction between the two (F_(80,2920)_ = 2.05, p = 0.09). Similarly, no effects were observed on maximum spiking, input resistance, or rheobase in prelimbic neurons (all n.s).

## Discussion

Our study reports substantial changes to the excitability of layer V pyramidal neurons in the mPFC when rats are trained to respond for rewards in two distinct conditions. Following acquisition of the RI task we observed fewer action potentials in prelimbic neurons in response to increasing current steps. This effect was significantly correlated with successfully withholding an inappropriate response, which suggests a cellular substrate for impulse control. Conversely, training with a cue that predicted imminent reward availability, but did not require the inhibition of a response, produced an increase in infralimbic excitability. These correlates indicate a dissociation between the adjoining sub-regions of the mPFC and suggest that they have distinct roles in the preparation and inhibition of actions in response to environmental cues.

We previously reported enhanced glutamatergic transmission in the prelimbic region after learning the RI task [12], an adaptation that appears in opposition to our current finding of decreased excitability in these neurons. Several other groups, however, have also reported plastic changes to intrinsic excitability concomitant with opposing adaptations to glutamatergic transmission. For example, cocaine withdrawal increased AMPA surface expression [22], [23] and decreased excitability [24], [25] of medium spiny neurons in the nucleus accumbens. Similarly, these cells show increased AMPA/NMDA ratio, but decreased excitability from youth to adulthood [26]. Excitability changes may occur through a homeostatic mechanism [27], akin to synaptic scaling [14], which would allow a neuron to remain within the limits of physiological firing rates, despite enhanced excitatory inputs.

Excitability was strongly correlated with performance in the final session of the RI task. Subjects at the lower end of this distribution (i.e. those making more premature responses), however, had excitability equivalent to the untrained rats (see Figure 3). Our previous study suggests that this level of performance correlates with elevated AMPA/NMDA ratios in these neurons [12]. Therefore, if synaptic scaling occurs during RI learning, we would expect to observe decreased excitability along with the increased synaptic transmission. This suggests that changes in excitability in these subjects are lagging behind the synaptic changes, much like their performance in the RI task.

To date, learning-induced modulations of intrinsic excitability have been reported with a number of paradigms including odor discrimination in the piriform cortex [28], [29], trace conditioning [30] and spatial learning [31] in the hippocampus, and delay conditioning in the cerebellum [32]. Fear conditioning and extinction provide the only evidence for experience-dependent modulation of intrinsic excitability in the mPFC [16]: infralimbic excitability decreased following conditioning with a shock-tone combination and extinction of the conditioned freezing response to the tone, by presenting the tone alone, restored excitability to pre-conditioning levels. Intriguingly, both fear conditioning and RI training inhibit the subject's movements: freezing in response to a tone after fear conditioning and withholding a response until an appropriate signal is presented in the RI task. The fact that both paradigms also decrease neuronal excitability supports the idea that mPFC activity is critical for the initiation or selection of actions [33]. This would also explain the close synchrony between mPFC and motor cortex activity in rats performing an impulse control task that is similar to the RI paradigm [3].

If mPFC neurons are directing actions, then changes to excitability in the prelimbic and infralimbic regions may reflect mPFC adaptations that control the initiation of responses. If true, increased AMPA/NMDA and decreased excitability in prelimbic neurons may control response inhibition by increasing the signal-to-noise ratio [34] of pyramidal neurons. That is, by decreasing excitability and increasing synaptic transmission, prelimbic neurons would be less likely to fire spontaneously, but respond preferentially to synaptic inputs. If mPFC activation promotes responding, then reducing spontaneous, ‘accidental’, action potentials would be immensely beneficial in preventing premature responses. This line of reasoning may explain previous reports of increased impulsivity and disrupted attention during stimulant withdrawal [35]–[37]. As described earlier, cocaine withdrawal increases excitability of mPFC neurons [18], and this elevation could counteract the training-induced reductions we observed after learning, leading to increased responding at an inappropriate juncture. This could have translational implications for the study of addiction, as impulsivity is a critical factor in predicting relapse [1], and suggests a mechanism for increased impulsivity during withdrawal from stimulants.

We designed our Operant Control condition to expose subjects to identical cues as the RI task, but with no opportunity to make a premature response. Fortuitously, this design allowed us to identify elevated infralimbic excitability after training with stimuli that reliably predicted lever insertion and reward availability. Importantly, training subjects with an equivalent, but unsignaled, delay had no effect on excitability. This suggests that the short audio-visual cue, which signaled imminent introduction of the lever, was driving the enhanced excitability. The stimulus would allow subjects to anticipate the lever's entry, and prepare their actions accordingly.

Enhanced excitability of infralimbic neurons in the Cue condition could reflect an interaction of this region with subcortical structures. The infralimbic cortex sends extensive projections to the basolateral amygdala (BLA) [38], [39], which has a role in cued-responding [40]. Neurons in the BLA respond to cues that have been paired with rewards [41], [42] and lesions to this region attenuate cue-induced responding [43]. Similarly, temporary deactivation of the BLA disrupts responding and the latency to lever press for a cued reward [44], [45]. Some authors [15] have argued that increased excitability has a permissive effect on further plastic changes. That is, processes like long-term potentiation may be easier to induce when the post-synaptic cell is more excitable. Therefore, it is possible that the changes we observed in infralimbic neurons may not be influencing behavior directly, but instead would allow the mPFC to adapt to changes in the predictive stimuli and in turn modulate its input to the BLA or other subcortical regions.

Alterations in the potassium conductances underlying AHPs have frequently been reported hand-in-hand with changes in neuronal excitability, including those associated with fear conditioning and extinction [16] in the mPFC. In this light, it was surprising that we observed no changes in AHP, but the most parsimonious explanation may be that the changes to excitability we observed in impulse control and cued responding are mediated by ionic channels not specifically measured in this study. Given the diverse array of channels that influence excitability [15], [46], identifying the specific changes could yield enormous therapeutic benefit for understanding impulse control and its dysfunction.

We identified two distinct neural correlates of learning to withhold a response: enhanced glutamatergic transmission and decreased intrinsic excitability in the prelimbic cortex. Although these findings are not comprehensive, they represent important clues for understanding the mechanism by which the mPFC directs actions. Moreover, building on these findings will allow a greater understanding of how the brain learns impulse control and, in turn, how it is disrupted in disorders such as ADHD and addiction.

## Materials and Methods

### Subjects

All the experiments were conducted in accordance with the Canadian Council on Animal Care guidelines for use of animals in experiments and approved by the Queen's University Animal Care Committee (2006-021). Thirty-nine male, Long-Evans rats (Charles River, QC, Canada), aged 21 post-natal days (PND) at the start of the experiment, were singly housed in standard polycarbonate cages on a reverse light-dark cycle (lights on at 7 pm). All testing was conducted during the dark cycle. During a 10-day acclimatization period, rats had free access to food (Lab Diet; PMI Nutrition International, Inc.) and water. Three days prior to training and for the remainder of the experiments, food was restricted to 120 min of daily ad libitum access, such that animals gained 10–15 g per week.

### Apparatus

Behavioral testing was conducted in operant boxes (26.5×22.0×20.0 cm), each housed in a sound-attenuating chamber (built in house). Each box was fitted with two retractable levers positioned on one wall. A food magazine was located between the two levers, which dispensed 45 mg dustless food pellets (BioServ, NJ). Signal lights were located 4 cm above each lever and the food magazine, and an indirect house light illuminated the entire chamber. A tone generator produced a sine wave 12–16 kHz, 80–90 db tone. A standard PC in an adjacent room controlled the equipment and was used for data collection (software written in-house using BASIC).

### Behavioral Training

Response inhibition was assessed using the RI task [12]. All behavioral training began after ten days of habituation to the housing conditions (PND 32). Initially, rats were magazine-trained for 1 day, receiving 20 sucrose pellets on a variable time 90-s schedule. Rats were then trained to lever press for food on a continuous reinforcement schedule. Only one lever was inserted into the chamber, with the lever assignment (left versus right) counterbalanced across animals and conditions. Lever assignment remained consistent for future stages of the experiment. A signal light above the assigned lever (lever light) was turned on throughout these sessions, except during delivery of the reward (1-s), which was paired with illumination of the signal light above the food magazine (magazine light). Training continued until the rat earned a minimum of 80 pellets in a 60-min session for 2 consecutive days.

Rats then progressed to the full RI task (Figure 1A). Trials progressed through an inter-trial interval (ITI), premature phase, and response phase. During the 10-s ITI, all lights were extinguished and the lever was retracted. During the premature phase (4-s), the lever was extended, the tone activated, and the house light illuminated. Lever presses during this period reinstated the ITI with no delivery of a sucrose pellet. If the rats did not respond during the premature phase, the trial progressed to the response phase (10-s), which was signaled by illumination of the lever light. A lever press during the response phase delivered a sucrose pellet, illuminated the magazine light for 1-s, and then initiated the next trial. If the response phase elapsed with no lever press, the lever retracted and the next trial was initiated. In each trial, responses were classified as ‘premature’, ‘correct’, or ‘omission’. Sessions were terminated once rats obtained 100 pellets or completed 200 trials, and each rat was trained for 8 sessions.

Separate groups of rats were trained in one of 4 control tasks. ‘Operant Control’ rats (Figure 1B; n = 6) underwent an identical training procedure, but the lever was withdrawn during the premature phase, preventing premature responses. Training in the ‘Cue’ condition (n = 5) was similar to the Operant Control condition with the exception that the illumination of the lights during the response phase and reward delivery were omitted (the tone/houselight signal was present during the premature phase but animals had no opportunity to respond as the lever was retracted). The premature phase cue was removed in the ‘No Cue’ condition (n = 7); subjects were trained with a 14-s ITI, and no other stimuli. Subjects in the ‘No ITI’ condition (n = 6) were trained with no ITI and no signals. For all control conditions, responses in each trial were classified as ‘correct’, or ‘omission’. Sessions were terminated once rats obtained 100 pellets or completed 200 trials, and each rat was trained for 8 sessions. Naïve controls (n = 7) were given an identical feeding schedule as other subjects, but did not receive any behavioral training.

### Preparation of brain slices

Within two hours of the final training session (at PND 43–50), rats were anesthetized with isofluorane, euthanized, and the brains were extracted for slice preparation. Coronal slices (250 µm) were prepared on a vibrating blade microtome in an ice-cold, oxygenated physiological solution containing (in mM) 126 NaCl, 2.5 KCl, 1.2 MgCl_2_, 2.5 CaCl_2_, 1.2 NaH_2_PO_4_, 25 NaHCO_3_ and, 11 D-Glucose. Slices were incubated in oxygenated physiological solution at 34°C for at least 60 min, then transferred to a holding bath for patch-clamp electrophysiology. During patch clamp recordings, slices were constantly perfused (1.5 ml/min) with physiological solution maintained at 34°C and equilibrated with 95% O_2_/5% CO_2_.

### Electrophysiology recordings

The mPFC was visualized and pyramidal neurons were identified by shape, and located using white matter landmarks. Whole-cell current clamp recordings were obtained from layer V neurons with borosilicate glass pipettes (1.5–2.5 MΩ tip resistance) containing (in mM) 130 D-Gluconic Acid, 20 KCl, 10 HEPES, 1 MgCl_2_, 1 EGTA, 2 MgATP, 0.3 GTP. Recordings were obtained with a Multiclamp 700B amplifier connected to a Digidata 1440A digitizer (Molecular Devices Scientific, Sunnyvale, CA). Data were collected and analyzed using Axograph X for windows (V 1.2, AxographX.com).

Neurons were initially voltage-clamped at −70 mV for 5 min to allow diffusion between intracellular fluid and the recording pipette. Recordings were then made in current-clamp configuration and measures of resting membrane (Vm) were obtained; experiments were terminated if resting membrane exceeded −60 mV.

To measure excitability, the number of action potentials (APs) generated in response to increasing current steps (50–2000 pA, 800 ms steps; 50 pA increments at 0.1 Hz) were computed while neurons were held near −70 mV. ‘Maximum Spikes’ was measured as the most APs observed in a cell at any current step. Input resistance was monitored throughout experiments with a 50 pA, 100 ms current step interleaved between the increasing current steps.

Afterhyperpolarization potentials (AHP) were measured with two protocols. Fast AHP (fAHP) were produced by a current step (800 ms, 0.1 hz) that reliably produced two action potentials. fAHP were calculated by subtracting the post-firing nadir from the firing threshold of the second AP. Medium and slow AHP (mAHP and sAHP) were produced by injecting twenty current steps (10 ms, 50 Hz), sufficient to generate an AP on each step. mAHP was calculated by subtracting the post-spike nadir from the pre-injection baseline and sAHP was calculated by subtracting the average potential 280 to 330 ms after the current step from the pre-injection baseline. To obtain a reliable measure, at least six (and up to 12) successful AHPs were averaged per cell.

### Statistical analyses

All statistics were analyzed using SPSS statistics (Version 19.0; IBM). Accuracy was assessed as the percentage of trials in which animals successfully inhibited lever pressing in the premature phase. This dependent measure was calculated as a percentage of correct response out of total trials with a response (100*(correct responses)/(premature+correct responses)). Latency (s) to lever press in the response phase was also compared. Behavioral data were analyzed using repeated measures analysis of variance (ANOVA) with Group as a between-subjects variable and Session as the within subject variable.

Electrophysiological data on action potentials were analyzed using repeated measures ANOVAs with one within-subjects factor (Current) and one between-subjects factor (Group). Simple effects ANOVAs were conducted at individual current steps if significant interactions (Group X Current) were present. The Greenhouse-Geisser correction [21] for significance was reported for all p values of within-subject effects (but uncorrected degrees of freedom are shown). Neuron properties were analyzed with a one-way (Group) ANOVA and post-hoc tests (Fischer's LSD) were conducted where appropriate. Linear regressions examined whether behavioural measures predicted electrophysiological properties, using adjusted R-squared (R^2^_adj_) values to determine the degree of the relationship.
